# Role of REM Sleep, Melanin Concentrating Hormone and Orexin/Hypocretin Systems in the Sleep Deprivation Pre-Ischemia

**DOI:** 10.1371/journal.pone.0168430

**Published:** 2017-01-06

**Authors:** Marta Pace, Antoine Adamantidis, Laura Facchin, Claudio Bassetti

**Affiliations:** 1 Center for Experimental Neurology (ZEN), Department of Neurology, Bern University Hospital, Bern, Switzerland; 2 Department of Neuroscience and Brain Technologies, Italian Institute of Technology (IIT), Genova, Italy; 3 Sleep-Wake-Epilepsy-Center, Department of Neurology, Bern University Hospital, Bern, Switzerland; 4 Division of Cognitive and Restorative Neurology, Department of Neurology, Bern University Hospital, Bern, Switzerland; Universidad Anahuac Mayab, MEXICO

## Abstract

**Study Objectives:**

Sleep reduction after stroke is linked to poor recovery in patients. Conversely, a neuroprotective effect is observed in animals subjected to acute sleep deprivation (SD) before ischemia. This neuroprotection is associated with an increase of the sleep, melanin concentrating hormone (MCH) and orexin/hypocretin (OX) systems. This study aims to 1) assess the relationship between sleep and recovery; 2) test the association between MCH and OX systems with the pathological mechanisms of stroke.

**Methods:**

Sprague-Dawley rats were assigned to four experimental groups: (i) SD_IS: SD performed before ischemia; (ii) IS: ischemia; (iii) SD_Sham: SD performed before sham surgery; (iv) Sham: sham surgery. EEG and EMG were recorded. The time-course of the MCH and OX gene expression was measured at 4, 12, 24 hours and 3, 4, 7 days following ischemic surgery by qRT-PCR.

**Results:**

A reduction of infarct volume was observed in the SD_IS group, which correlated with an increase of REM sleep observed during the acute phase of stroke. Conversely, the IS group showed a reduction of REM sleep. Furthermore, ischemia induces an increase of MCH and OX systems during the acute phase of stroke, although, both systems were still increased for a long period of time only in the SD_IS group.

**Conclusions:**

Our data indicates that REM sleep may be involved in the neuroprotective effect of SD pre-ischemia, and that both MCH and OX systems were increased during the acute phase of stroke. Future studies should assess the role of REM sleep as a prognostic marker, and test MCH and OXA agonists as new treatment options in the acute phase of stroke.

## Introduction

Ischemic stroke is one of the major causes of death and permanent disability worldwide [1, 2]. Recombinant tissue-type plasminogen (rtPA) is the standard treatment for acute ischemic stroke [3]. However, even if randomized trials have demonstrated that more patients show good outcomes in 50–70% of cases when treated with rtPA [4, 5], this therapy, unfortunately, is only available to less than 10% of the patients [6], due to the narrow therapeutic window (<4.5 h) and the increased risk of intracranial haemorrhage [5].

These limitations have emphasized the need for alternative therapies. Most of these therapeutic approaches have focused on protecting neurons from the main pathogenic mechanisms causing ischemic injury, such as excitotoxicity, oxidative stress, inflammation or apoptosis [7]. These neuroprotective agents have shown good results in animal models, however, when they have been tested on humans they have failed dramatically [8].

Current efforts to find alternative therapies are working towards understanding the brain’s endogenous protective mechanisms [9], including preconditioning. Preconditioning is a procedure by which a noxious stimulus, near to but below the damage threshold, induces an adaptive response that protects against subsequent ischemia [10].

Several stimuli have been used as preconditioning before ischemia including total sleep deprivation (SD pre-ischemia). In fact, it has been observed that SD pre-ischemia confers protection against subsequent ischemic damage by significantly reducing infarct volume after 7 days from ischemic stroke [11, 12]. Moreover, significant reductions in inflammatory response and apoptotic processes that strongly and negatively affect stroke outcome were observed [13, 14]. Additionally, our group had previously observed that SD pre-ischemia animals showed a significant increase in the amount of total sleep during the first 24h following ischemia. This suggests that the increase in the total amount of sleep during the acute phase of stroke positively modulates functional recovery [11]. Consistent with the hypothesis that sleep is essential for functional recovery, pharmacological enhancement of slow wave sleep after ischemia showed a positive outcome in animal models of stroke [15, 16].

Sleep-reduction/fragmentation is very common after ischemic stroke, affecting at least, 20% to 40% of stroke patients, and is linked with poor functional outcomes [17–20]. These sleep disturbances following ischemic stroke may involve both stages of sleep, rapid eye movement (REM) and non-rapid eye movement (non-REM) [17]. Particularly, the reduction of REM sleep has been associated with negative functional recovery [21, 22]. Conversely, a recent study conducted on chronic ischemic stroke patients showed that the more time the patient spent in REM sleep, the more offline motor learning increases [23]. However, the specific role of REM sleep on ischemic stroke, and how it may affect patient outcomes remains unknown.

A recent study published by our group [24] observed an increase of two genes, melanin-concentrating hormone (MCH) and Orexin/Hypocretin (OX), (see Box 1 on Physiological roles of OX and MCH neurons), in SD pre-ischemia animals and not in ischemia animals alone after 3 days from ischemia. A body of evidence has already shown an early involvement of OXA in the pathophysiology following ischemic stroke by identifying several mechanisms of action [25–27]. However, to date the involvement of MCH and OX in the neuroprotective effect elicited by SD is elusive; this study describes for the first time a possible role or association between MCH and ischemic stroke.

**BOX 1. Physiological roles of Orexin/hypocretin (OX) and Melanin-Concentrating Hormone (MCH) neurons**

Neurons containing OX or MCH are mainly localised in the lateral hypothalamus. Each constitutes a separate and distinct neuronal population, even if they project to similar target areas in the brain according to their respective receptors [64–67]. The main differences and similarities between these two neuropeptides are briefly discussed below OX neurons synthetize two different neuropeptides: Orexin/hypocretin-A (OXA) and Orexin/hypocretin-B (OXB). OX effects are mediated by two subtypes of OX receptors, which are OX Recepor-1 (OX1R) and OX Recepor-2 (OX2R) that are differently expressed in the brain. Particularly, OXA may satisfy its physiological functions by binding both OX1R and OX2R, whereas OX-B signals mainly act through OX2R [68]. Physiological functions of the OX peptides include the stabilization of wakefulness [69, 70], energy expenditure [71], behavioural responses to food reward and addiction [72], and lastly increase food intake. It has also been observed that OXA release is stimulated by low glucose and inhibited by high glucose [73]. Finally, it has been shown that OXA is involved in the regulation of cardiovascular responses [74] and thermoregulatory systems [71], as well as inflammation, suggesting an anti-inflammatory function in neuro-inflammation diseases [62] and also inhibits apoptosis [61]. Recently, a consistent body of evidence has highlighted a possible neuroprotective role of OXA in ischemic stroke by either modulating inflammation [27] or modulating post-ischemic glucose intolerance [25, 75].

MCH exerts its action through the MCH1 receptor (Mchr1) and MCH2 receptor (Mch R2), although the latter is not functional in rodents [76, 77]. As widely described, MCH works in a complementary or even opposite manner to OX. Notably, MCH neurons play opposing roles in the regulation of sleep-wake cycle. Indeed, MCH promotes sleep, particularly MCH neurons fire during REM sleep [78]. In contrast to OX, MCH promotes energy conservation and the activation of MCH neurons are regulated by elevated glucose levels [79]. Finally, MCH supports depression and anxiety, while OX increases the reinforcing properties of ingested substances [80]. However, MCH, as well as OX, are orexigenic peptide, meaning that both stimulate food intake and promote the consumption of palatable or caloric food [80].

This study aims firstly at defining the role of REM sleep on stroke, and if REM sleep correlates with positive outcomes after ischemic stroke, and thus, if it may be used as a prognostic marker. The second aim is to investigate if both the MCH and OX systems are associated with the pathophysiology of stroke and with the beneficial effect elicited by SD. To test the association, a time course of the expression of genes related to these systems was performed. Finally, as MCH and OX systems are involved in the regulation of the sleep-wake cycle, although in opposite ways, we aim to understand whether the expression of genes related to these systems following ischemia are particularly tied to the sleep-wake cycle.

## Materials and Methods

Male Sprague-Dawley rats (n = 112), 9–11 weeks old and weighing 300 ± 50 g at the time of surgery, were used in this study. They were housed under 12-h light/dark cycle (light on 08:00–20:00) with ambient temperature at 22 ± 0.5°C. Food and water were provided ad libitum. All animal procedures were approved by the Animal Research Committee and the Veterinary Office of the Canton of Bern, Switzerland.

### Experimental design

**Fig 1:**
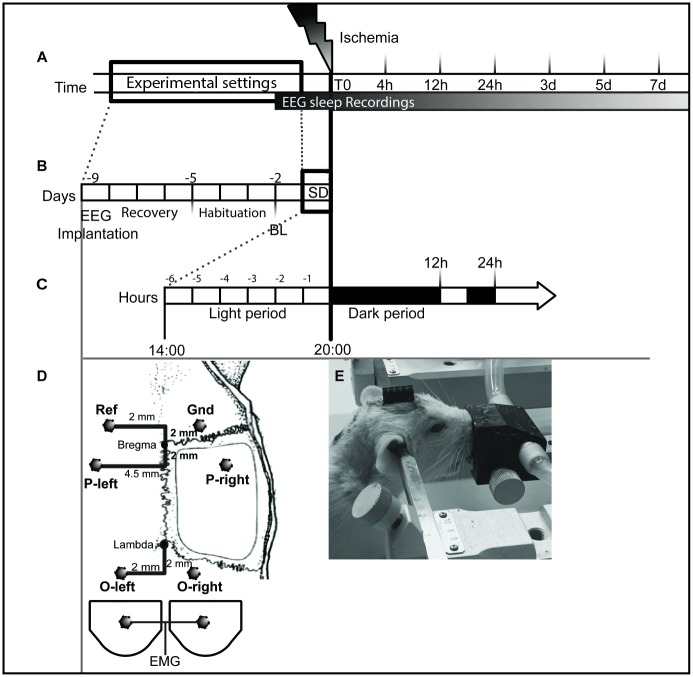
Schematic of the experiment design. **(A)** Design for the sleep architecture analysis and for the time course of gene expression of MCH and OX systems. Ischemia surgery was performed on day 0, and then the rats were sacrificed at 4, 12 and 24 hours (acute phase) and 3, 4 and 7 days (chronic phase) following ischemic surgery. Baseline was recorded 2 days before ischemia for 24h; 12h dark and 12h light. Rats subjected to SD were also recorded over 6h of SD. The EEG/EMG recordings were stopped when animals were sacrificed; represented by each time point **(B)** Design for sleep architecture. Rats were implanted with EEG/EMG electrodes and then allowed to recover for 4 days, and then connected to a flexible cable and swivel and habituated for 3 days with a cable, before EEG/EMG recording. **C)** Design for SD interventions. SD was performed by gentle handling during the last 6 h of the light period; from 14:00 to 20:00. And ischemia/sham surgery was performed immediately after; at the beginning of the dark period, during either stroke or sham surgery EEG/EMG recordings were not performed **(D)** Not-to-scale representation of the placement of the screw electrodes over the parietal cortex and the cerebellar cortex (dark circles), Ref. = reference and Gnd = ground. EMG was bilaterally placed in the neck muscle using wire electrodes **(E)** An anesthetized rat fixed to the stereotaxic frame with EEG/EMG plug fixed to the skull with dental cement. Experimental groups: i. SD_IS (n = 6); ii. IS (n = 6); iii. SD_Sham (n = 4); and iv Sham (n = 6). Electroencephalogram (EEG); Electromyogram (EMG).

Rats were randomly assigned to the following experimental groups: (i) sleep deprivation followed by ischemia (SD_IS); (ii) ischemia (IS); and (iii) sham surgery (Sham). Each experimental group comprised 6 animals, which were sacrificed at several time points following ischemia: 4; 12; and 24 hours (acute phase) and 3, 5 and 7 days (chronic phase), (Fig 1A). Overall, 18 animals were analysed at each time point except at 3 days, when an additional experimental group underwent sleep deprivation followed by sham surgery (SD_Sham n = 4). SD_Sham group was added only to assess the effect of SD over a longer period of time.

### Sleep deprivation procedures

Total sleep deprivation was performed by gentle handling techniques, consisting of introducing novel objects into the cage, knocking or shaking the cage when behavioral signs of sleep were observed. Animals were subjected to SD during the last 6h of the light period, and ischemia/sham surgery was performed immediately after, at the beginning of the dark period (Fig 1C) as previously described [11, 12]. Polysomnographic recordings were performed during the whole period of total SD.

### Surgical procedures

#### Implantation of EEG and EMG electrodes

Rats were anesthetized with 1.5%–2.5% isoflurane in oxygen and surgically implanted with electroencephalogram/electromyogram (EEG/EMG) electrodes for sleep-wake recording. Mini-screw electrodes were implanted bilaterally in the parietal cortex (coordinates: 2 mm posterior of the bregma and 4.5 mm lateral to the midline in the left and right parietal skull) and cerebellar cortex (coordinates: 2 mm posterior of the lambda and 2 mm lateral to the midline in the left and right frontal skull) (Fig 1D). EMG was recorded by 2 stainless steel wires inserted bilaterally into the neck muscles (Fig 1D). Following surgery, all animals received paracetamol (200 mg/kg; twice a day; PO; Tempra) and enrofloxacine (10mg/kg; once a day; SC; Baytril) for three days after surgery. Animals were housed individually in their home cages for a recovery period of 4 days, and then each rat was connected to a flexible cable and swivel (Plastics One) that allowed free movement within the chambers, and habituated for 3 days with a cable, before EEG/EMG recording (Fig 1B and S1 Fig). Rats were recorded continuously for 24 h for a baseline (12h:12h dark-light cycle), 6h during total SD, and over the time until animals were sacrificed (Fig 1A and 1B). Notably EEG/EMG recording was not performed during either the sham or stroke surgery.

#### Ischemic stroke surgery

Stroke was induced by the three-vessel occlusion method (3Vo) [28], which predominantly affects the primary somatosensory cortex, avoiding thalamic, hypothalamic, hippocampal, and midbrain damage [29]. We used this model because the relative infarct volume in relation to brain size corresponds to the majority of human strokes. Moreover, this is a reproducible model with low mortality. 3Vo consists of the permanent occlusion of the distal middle cerebral artery (MCA) and the ipsilateral common carotid artery (iCCA), whereas the contralateral CCA (cCCA) was transiently occluded for 60 min with an aneurysm clip. Stroke surgery was performed under general anesthesia with 2% isoflurane in oxygen. A small piece of skull overlying the MCA was removed and the dura mater was retracted. The MCA and its three main branches were occluded by bipolar electro coagulation. Body temperature was maintained between 36.5±0.5°C by a heating pad. Sham-operated rats were subjected to the same procedure as well as the same time exposure for anaesthesia (approximately 90 minutes) except for the occlusion of the MCA and the CCA. After surgery rats were returned to their cages and EEG/EMG were resumed until the end of the experiment (for time points see Fig 1). To assess animal health and body condition, all animals were monitored until the end of the experiment for the following parameters: body weight chenges, appetite, gait, posture and attitude (i.e bright, alert, responsive or burrowing or hiding, quiet but rouses when touched and finally, no cage exploration when lid off, burrows/hides, may vocalize or be unusually aggressive when touched).

### Electrophysiological data analysis

The EEG/EMG signals were amplified (Grass Instruments, USA), and digitized at a sampling rate of 100 Hz and collected on a PC using VitalRecorder (Kissei Comtec Co. Ltd, Japan). EEG signals were filtered at 0.3 Hz (low pass filter) and 0.1 KHz (high pass filter), respectively, whereas EMG, at 1000 Hz. The polysomnographic recordings were visually scored offline-using SleepSign software (Kissei Comtec Co. Ltd, Japan), per 10 second epoch window, as wakefulness, non-REM sleep or REM sleep as previously described [11]. Scoring was performed by a single observer, blinded to rat identity. The percentage of time spent in wakefulness, non-REM and REM was determined for each hour. The total number and length (seconds) of REM sleep bouts were assessed across the 24h of baseline and for the following three days after ischemic stroke. REM sleep bout numbers were counted as each continuous episode of the REM sleep state. REM sleep bout length was calculated by the amount of time spent in separate bouts. Two EEG signals come from ipsilateral and contralateral hemispheres to the lesion and muscle (EMG) were recorded, although, the behavioral states were scored using the contralateral hemisphere (healthy hemisphere). However, when the signal was unclear, affected by artefact movement or electrical noise, the recordings from the ipsilateral hemisphere were consulted, and eventually tagged and excluded from subsequent analyses. Polysomnographic recordings started immediately after ischemic or sham surgery, although the analysis of the first 30/40 minutes was excluded because unusual spikes due to isoflurane anaesthesia were observed in all animals.

### Brain collection and infarct volume evaluation

At the end of the experiment, rats were decapitated while deeply anesthetized (Isoflurane 5%) and brains dissected and frozen immediately in dry ice. For infarct volume evaluation, coronal sections of 20 μm were cut on a cryostat at six predefined levels (L) with 1 mm interval (L-1: 2.7 mm; L-2: 1.7 mm; L-3: 0.7 mm; L-4: −0.3 mm; L-5: −1.3 mm and L-6: −2.3 mm from bregma) and stained with cresyl violet and digitized [30, 31]. The remaining tissue between these sections was cut at 50 μm and ischemic and contralateral hemispheres collected separately and stored at −80°C for gene expression analysis. The infarct area was measured for each level by the public-domain ImageJ program (http://imagej.nih.gov/ij). All photographs were analysed by two independent observers blinded to rat identity. Correction of the infarct volume for edema was first calculated by subtracting the size of the undamaged area in the stroke hemisphere from that in the intact hemisphere and then converted with the known distance between each level.

### Gene expression analysis by quantitative real-time polymerase chain reaction (qRT-PCR)

RNA was isolated separately from the ischemic and contralateral hemispheres from the 50 μm sections by the Trizol method (Sigma Aldrich, Midtown-St Louis, MO, USA) according to the manufacturer’s instruction [32]. RNA concentration was then determined by a NanoDrop 2000c spectrophotometer. The complementary DNA was obtained from up to 2 mg of total RNA by using a high-capacity RNA-to-cDNA kit (Invitrogen) and stored at -20 C°. TaqMan Gene Expression Assay (Life Technologies, Carlsbad, CA, USA) was used to analyse the gene expression of the MCH-system and OX-system. Notably, the analysis of the MCH-system included the assessment of the precursor of MCH (*Pmch*) and its receptor MCH receptor 1 (*Mchr1*). Whereas, the analysis of the OX-system comprised the assessment of OX-A (*OxA*) since it has been already observed to play a role in post-ischemic stroke and its two receptors, OX receptor-1 (*Ox1R*) and OX receptor-2 *Ox2R* (see Table 1 for TaqMan assay references sequencing). Additionally, we investigated the gene expression of brain-derived neurotrophic factor (*Bdnf*) since it is involved in neuroplasticity and neurogenesis process [33]. Reactions were performed in triplicate using AB 7900HT fast real-time PCR system (Applied Biosystems). The relative level of mRNA was calculated as follows: mRNA = 2^(-ΔCT experiment rat−ΔCT sham rat)^, where ΔCT = (C_T. target_−C_T. Gapdh_) [15].

**Table 1:** List of Taqman assays used for the qRT-PCR analysis.

Gene	RefSeq
*Gapdh* (Glyceraldehyde 3-phosphate dehydrogenase used as endogenous control)	Rn01775763_g1
*Pmch* (Precursor of melanin concentrating hormone (MCH))	Rn00561766_g1
*Mchr1* (MCH receptor 1)	Rn00755896_m1
*Oxa* (Orexin (OX)-A/Hypocretin 1)	Rn00565995_m1
*Ox1R* (OX receptor 1)	Rn00565032_m1
*Ox2R* (OX receptor 2)	Rn00565155_m1
*Bdnf* (Brain-derived neurotrophic factor)	Rn02531967_s1

### Statistical analysis

Gaussian distribution of values was tested with homogeneous variance (Levene test). Data were presented as mean ± standard error of the mean (SEM). Infarct volume was assessed by unpaired t-test. Sleep architecture across the acute phase was tested by paired t-test. Sleep changes over the sub-acute and chronic phase after interventions was evaluated by repeated measures ANOVA, whereas two-way ANOVA (factors: group and time) was used for analysing REM sleep bouts. Gene expression across groups at several time points was assessed by one-way ANOVA. Whenever ANOVA statistical significance was achieved, Tukey’s multiple post hoc contrasts were performed to determine group-wise comparison. Pearson correlation was performed in order to assess a link between the percentage of REM sleep across the acute phase after stroke and the infarct volume assessed after 7 days from stroke. GraphPad Prism6 (GraphPad Prism Software, Inc) was used for statistical analysis. Type I error α was set at 0.05 (p < 0.05).

## Results

### Infarct volume analysis

In order to confirm the neuroprotective effect of SD pre-ischemia, infarct size was evaluated in both IS and SD_IS groups at several time points: 12h, 24 h, 3, 4 and 7 days following experimental ischemia. Animals subjected to sham surgery did not reveal any brain lesions.

**Fig 2:**
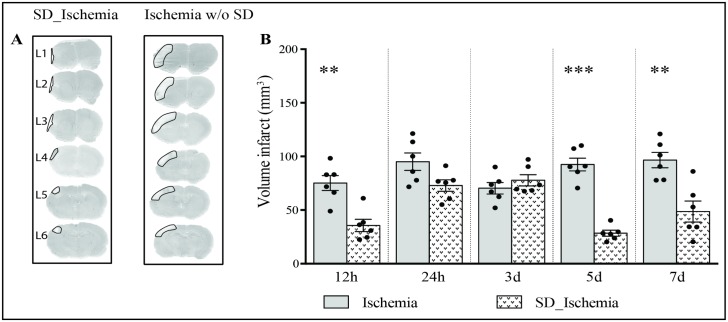
Effects of sleep deprivation (SD) pre-ischemia on the infarct volume. Lesion volumes corrected for edema were calculated by cresyl violet staining at 12 and 24 hours and 3, 5 and 7 days after ischemic surgery are displayed on the x-axis (see S2 Fig for infarct volume assessed without edema correction and S1 and S2 Tables for data set). **(A)** Representative sets of brain sections from a rat subjected to 6h of SD pre-ischemia (left panel), and rats subjected to ischemia without SD (right panel). The infarct areas are delineated by a thin black line. L1 is at 2.7 mm anterior to bregma, and the interval between each level is 1 mm (see methods). **(B)** Infarct volume (mean ± SEM) assessed at 12 and 24 hours and 3, 5 and 7 days after interventions (n = 6 per group) were analysed by unpaired t-test. Dots represent infarct volume of each animal during each time points. Asterisks (*) indicate a statistical difference between groups, **p ≤ .01; *** p ≤ .001.

SD_IS group showed a significant reduction in infarct volume at 12h (SD_IS: 35.62 ± 5.68 vs. IS: 75.27 ± 6.92 mm^3^; t(10) = 4.42, p = .001), 5 days (SD_IS: 28.4 ± 2.77 vs. IS: 92.52 ± 5.95 mm^3^; t(10) = 9.75, p = .0001) and 7 days (SD_IS: 48.65 ± 9.74 vs. IS: 96.66 ± 7.19 mm^3^; t(10) = 4.42, p = .002, Fig 2B) following ischemia. No significant reduction in infarct volume was observed at 24h (SD_IS: 81.25 ± 6.37 vs. IS: 95.10 ± 8.12 mm^3^, t(10) = 1.34, p = .20) and at 3 days (SD_IS: 70.35 ± 5.33 vs. IS: 77.79 ± 5.23 mm^3^, t(10) = 0.99, p = .34, Fig 2B) following ischemia. At 4h infarct volume was not assessed because the ischemic lesion was not evident by cresyl violet staining.

### Sleep-wake cycle architecture

#### Sleep EEG at the baseline

At the baseline (BL) recordings we did not observe any statistical differences concerning the circadian distribution of sleep between animals that were subsequently randomly divided into the four experimental groups: IS (n = 6); SD_IS (n = 6); Sham (n = 6) and SD_sham (n = 4) groups. For sleep EEG analysis animals sacrificed at 7 days were analysed, except for the latter group where animals sacrificed at 3 days were used. To make correct comparisons respect to the control group (SD-Sham) sleep EEG changes were investigated till 3 days after interventions. Data pooled from the 4 experimental groups shows an increase in sleep time at BL during the light period of the light/dark cycle: animals slept for approximately 57% of the recorded 12 hours (non-REM sleep: 47.65% ± 1,57%; REM sleep: 10.10% ± 0.37%) and approximately 22% during the dark period (non-REM sleep: 16.53% ± 0,81%; REM sleep: 5.46% ± 0.38%). Animals that were subjected to SD interventions (SD_IS and SD_Sham) were kept awake for approximately 99.71% ± 0.50% of the 6h-sleep deprivation procedure (non-REM sleep: 0.29% ± 0.50%; REM sleep:0%).

#### Changes in sleep EEG during the first 24h after interventions

**Fig 3:**
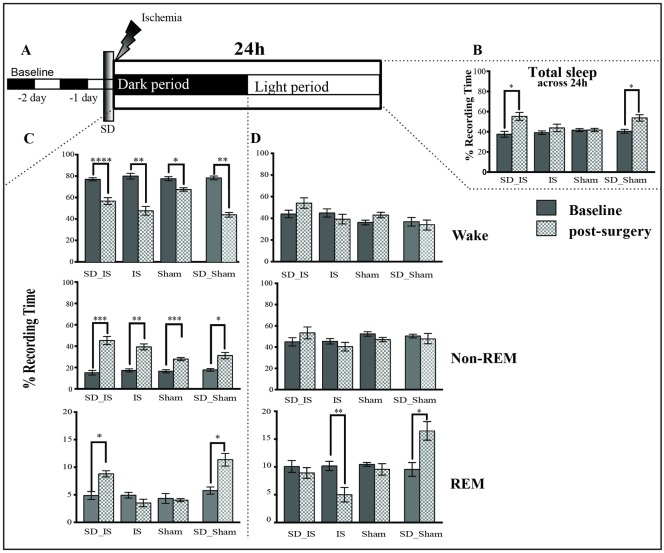
Changes in sleep EEG during the first 24h after interventions. **(A)** The time line of the experiment. Sleep was analysed at the baseline before SD and after 24h from either ischemia or sham surgery; 12h dark,12h light periods. **(B)** The amount of total sleep (mean ± SEM) is represented by vertical bars. Dark bars indicate the baseline time, which was defined as the percentage of time spent in each state across 24h of recording baseline. White bars indicate post-surgery times across 24h after interventions. **(C)** Total, wake, non-REM and REM sleep (mean ± SEM) were analysed separately over the dark period. **(D)** Total, wake, non-REM and REM sleep (mean ± SEM) were analysed separately over the light period. Comparison with corresponding baselines was performed with paired t-tests (see S3 and S4 Tables for data set). Stars denote post-surgery times that differed significantly from baseline time and specific p values are shown. * P ≤ .05; **p ≤ .01; *** p ≤ .001; **** p ≤ .0001. Experimental groups: i. SD_IS (n = 6); ii. IS (n = 6); iii. SD_Sham (n = 4); and iv Sham (n = 6).

During the first 24 hours (acute phase) of either stroke or sham surgery the changes in the amount of total sleep (including both non-REM and REM sleep) showed a significant increase exclusively in animals subjected to SD interventions compared to their baseline across the 12h:12h dark-light cycle. Indeed an increase of 15–20% in the amount of total sleep was observed in both groups SD_IS (BL: 37.53% ± 2.88% vs. post-surgery: 55.29% ± 3.90% mm, t(5) = 3.59, p = .01) and in the SD_Sham groups (BL: 40.45% ± 1.53% vs. post-surgery: 53.60% ± 1.74% t(3) = 3.58 p = .0009, Fig 3B).

During the dark period that immediately followed surgery (i.e. active phase in rats), the percentage of non-REM sleep was significantly increased (SD_IS p = .0001; IS p = .001; SD_Sham: p = .03; Sham p = .0009, Fig 3C), whereas the amount of wakefulness (SD_IS p = .0001; IS; SD_Sham: p = .004; Sham p = .01, Fig 3C) was significantly decreased in all conditions when compared to their baseline. REM sleep was significantly increased only in the two groups subjected to SD (SD_IS p = .02; SD_Sham: p = .04, respectively), whereas the IS and sham groups did not differ compared to the baseline (Fig 3C).

During the light period that started 12h after interventions (i.e., resting phase in rats), non-REM sleep and wakefulness were unchanged in all conditions compared to baseline (Fig 3D). Interestingly, REM sleep was significantly reduced only in the IS group (BL: 10.18% ± 1.30% vs. post-surgery: 5.35% ± 0.83%, t(5) = 5.35, p = .003; Fig 3D) and increased in the SD_Sham group (BL: 9.66% ± 0,77% vs. post-surgery: 16.91% ± 3.02%, t(3) = 3.23, p = .04; Fig 3D), whereas it was not statistically different in the SD_IS and Sham groups compared to the baseline (Fig 3D).

**Fig 4:**
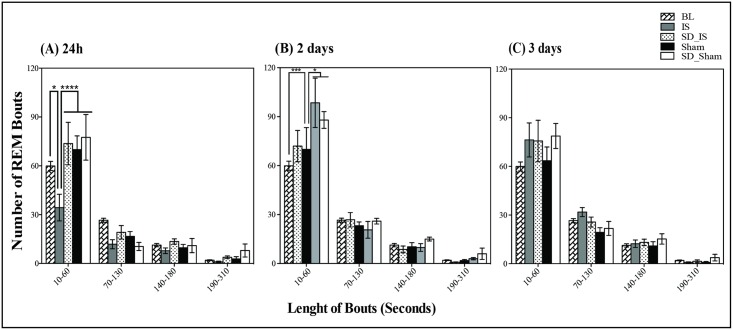
The number and length of bouts of REM sleep. Each number of REM bouts as a function of sleep period length (in seconds) is shown over the first 24h **(A)**; 2 days **(B)** and 3 days **(C)** after interventions; sleep deprivation (SD) and either ischemia and sham surgery and during the baseline time. The daily number of REM sleep bouts during baseline accounts for the control values (n = 6 baseline values belonging to each animal randomly assigned to the experimental groups were averaged for each condition). The figure represents the average (±SEM) number of sleep periods of a specific length in the following experimental groups: i. SD_IS (n = 6); ii. IS (n = 6); iii. SD_Sham (n = 4); and iv Sham (n = 6). Statistical analyses were performed by two-way ANOVA (factors: “group×length of the bout”) and post hoc analysis, with Tukey’s multiple comparison tests run afterward. a: p ≤ .01, IS group vs. baseline; SD_IS, SD_Sham and Sham groups. b: p< .0001, SD_IS group vs. baseline, IS and Sham groups.

In addition, the duration and frequency of REM sleep bouts were assessed during the acute phase after stroke and sham surgery. The number of short bouts of REM sleep (from 10 to 60 seconds), was markedly reduced in the IS group (two-way ANOVA: F (12, 84) = 2.48 p = .007; Fig 4A “group×length of the bout” interaction effect) compared to all other groups investigated and compared to the baseline. Notably, the number of short bouts of REM sleep in the SD_IS group was not statistically different when compared with the baseline values and with both sham groups.

**Fig 5:**
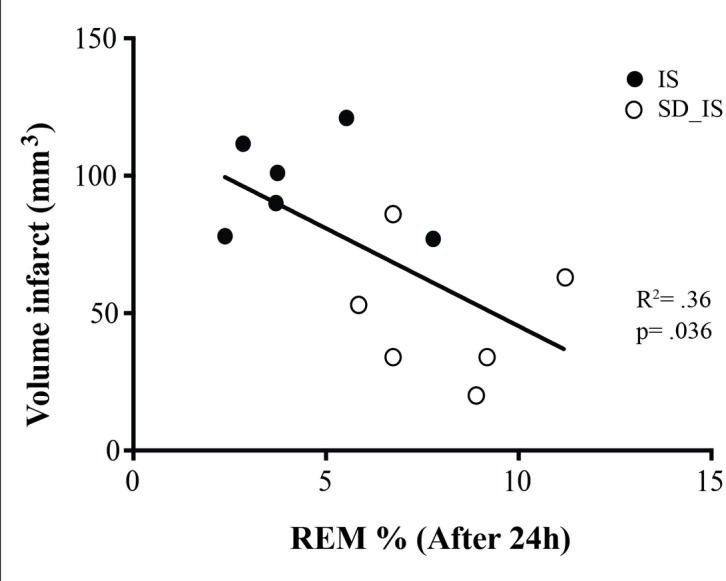
Correlation of the amount of total REM sleep phase during the first 24h after ischemic surgery with the infarct volume assessed at 7 days in 12 rats. REM sleep values as a percentage of total recording time, including both the light and dark periods (see S5 Table for data set).

Finally, to investigate how sleep and in particular the changes of REM sleep during the acute phase of ischemia (first 24h after ischemia) influence infarct volume, correlation of REM sleep with infarct volume was computed. The percentage of REM sleep recorded during the first 24h after ischemic surgery was negatively correlated with the infarct volume measured in the 12 rats (n = 6 rats belonging to the SD_IS group and n = 6 rats belonging to the IS group) which underwent ischemia (Pearson's R^2^ = .36, p = .036, Fig 5).

#### Changes in sleep EEG during 2 and 3 days after interventions

**Fig 6:**
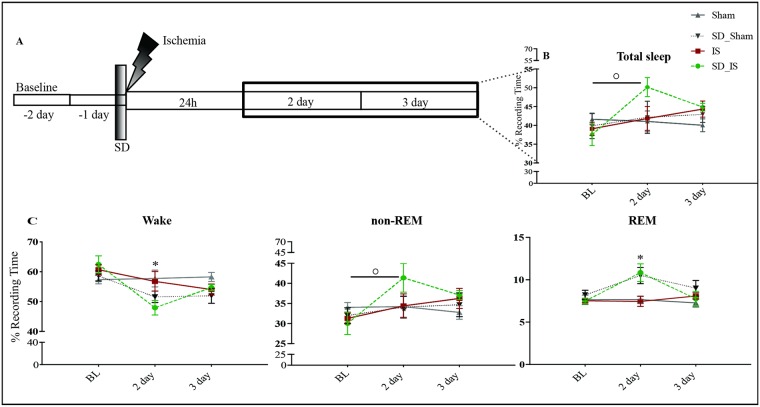
Changes in sleep EEG during the 2 and 3 days after interventions. **(A)** The timeline of the experiment. Sleep was analysed at the baseline and over the following 2 and 3 days. The baseline values was given by the percentage of time spent in each state across 24h of baseline recording. **(B)** The amount of total sleep (mean ± SEM) for each experimental group at the BL; 2 day and 3 day post-recovery (i. SD_IS (n = 6); ii. IS (n = 6); iii. SD_Sham (n = 4); and iv. Sham (n = 6)). Time (baseline, 2 and 3 days) is displayed in the x-axis. **(C)** Total, wake, non-REM and REM sleep (mean ± SEM) were analysed separately over the baseline and 2 and 3 days after interventions (see S3 Table for data set). Statistical analyses were performed rANOVA (factors: group and time) and post hoc analysis, with Tukey’s multiple comparison tests run afterward. Asterisks (*) indicate a statistical difference between groups (*P ≤ .05), whereas dots (°) indicate a statistical difference between days within the same group (°°P ≤ .05).

A repeated-measures analysis of variance revealed a significant effect of time on the total amount of sleep, (F(2, 36) = 3.81, p = .032 “time” Fig 6B), and the same was observed for non-REM sleep (F(2, 36) = 7,49 p = .001 “time” Fig 6C). Conversely a significant interaction between the two factors: treatment × time was observed for the amount of REM sleep (rANOVA: F(6, 36) = 2.66 p = .030 Fig 6C). Indeed, REM sleep was increased in both groups, which underwent SD (SD_Sham and SD_IS groups) at 2 days following interventions compared to IS and Sham groups.

Instead, wakefulness was significantly decreased in the SD_IS group at 2 days relative to IS and Sham groups (rANOVA: F(6, 36) = 3,41 p = .009 group x time” interaction effect Fig 6C).

The duration and frequency of REM sleep bouts were also assessed at 2 and 3 days after interventions. Interestingly, the number of short bouts of REM sleep (from 10 to 60 seconds) was still increased in the SD_IS group (2 days after stroke when compared to the baseline values, IS and Sham groups, as detected by two-way ANOVA (F (3, 84) = 135, p = .0001 “group” Fig 4B). At 3 days, the number of REM sleep bouts did not show any statistical differences between groups (Fig 4C).

### Gene expression analysis

**Fig 7:**
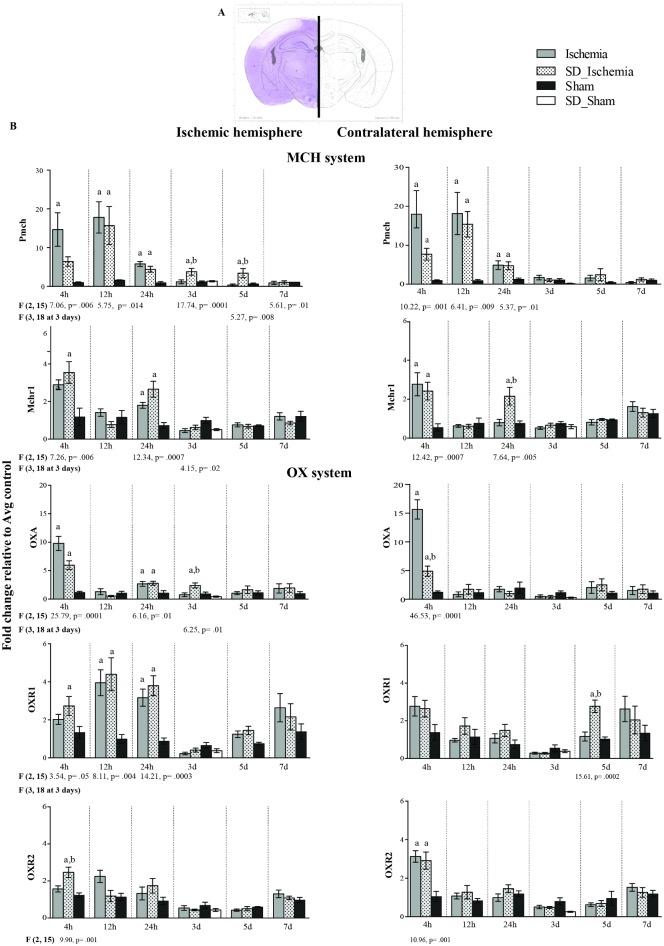
Time course of gene expression of the precursor of MCH (*Pmch*) and MCH1 receptor (*Mchr1*) in the ischemic and contralateral hemispheres. **(A)** An example (is at the level of bregma -1.82 mm on the rat brain atlas by Paxinos and Watson, which accounts for L6) of the six levels of the coronal section of rat brain dissected into two parts, ischemic and contralateral hemispheres for gene expression analysis by qRT-PCR (see methods for more detils). The white area in the left hemisphere displays the distribution of ischemic damage in the somatosensory cortex. **(B)** Time course of gene expression of *Pmch* and *Mchr1* at several time points (i.e. 4,12 and 24 hours and 3,4 and 7) after interventions are displayed on the x-axis (see S6 Table for data set). **(C)** Time course of gene expression of *OxA*, *Ox1R* and *Ox2R* at several time points (i.e. 4,12 and 24 hours and 3,4 and 7) after interventions are displayed on the x-axis. Gene expression (mean ± SEM) was assessed by qRT-PCR in rats belonging to the 4 experimental groups: i. SD_IS (n = 6); ii. IS (n = 6); iii. SD_Sham (n = 4); and iv Sham (n = 6). *Gapdh* was used as the reference gene. The ΔΔCt method was used to determine the fold change in gene expression. Statistical analysis was performed by one-way ANOVA (see F values and p values) and post hoc analysis with Tukey’s multiple comparison test was run afterwards. a: p< 05 when compared to sham group; b: p< 05 when compared to IS group; c: p< 05 when compared to SD_IS group and d: p< 05 when compared to SD_Sham group.

To test for an association of MCH and OX gene expression with the protective effect of sleep deprivation, time course of gene expression of *Pmch* and *OxA* and their receptors *Mchr1*, *Ox1R* and *Ox2R* were analysed at 4, 12, 24 hours and at 3, 5, 7 days following ischemia in both ischemic and contralateral hemispheres (Fig 7A). Both hemispheres were investigated 1) to confirm that these genes are indeed ischemia-related, and 2) to uncover any potential bilateral effects. Additionally, a gene expression of *Bdnf* was performed in all groups investigated at several time points, however no differences were found among groups over the time (see S3 Fig).

### MCH-system

#### Time course of precursor of MCH (*Pmch*)

Both groups that underwent ischemia (IS and SD_IS) showed a significant increase of *Pmch* during the acute phase of stroke (from 4h to 24h) in both hemispheres compared with the Sham group (see Fig 7B, for relative F values and p values). Moreover, the *Pmch* level was still increased exclusively in the SD_IS group in the lesioned hemisphere at 3 days (F (3, 18) = 5.27, p = .008) and at 5 days (F (2, 15) = 5.61, p = .01) (Fig 7B). Conversely, the *Pmch* level declined in the IS group from 24 to 7 days following ischemia, in both hemispheres, displaying the same level as the Sham group. At 7 days, *Pmch* did not differ in either groups or hemispheres (Fig 7B).

#### Time course of MCH1 receptor (*Mchr1*)

As observed for *Pmch* its receptor *Mchr1* was also increased during the acute phase of ischemia (at 4h and 24h following ischemia) in both groups which, underwent ischemia (IS and SD_IS groups) compared to Sham group. Notably, *Mchr1* was increased in both hemispheres of the SD_IS group (see Fig 7B, for relative F values and p values) whereas, in the IS group at 24h *Mchr1* was significantly increased only in the lesioned hemisphere (see Fig 7B, for relative F values and p values). Additionally, *Mchr1* did not show any statistical differences between groups at later time points, as was observed for *Pmch*.

### OX-system

#### Time course of Orexin (OX)-A/hypocretin-1 (*OxA*)

*OxA* was increased during the acute phase of ischemic stroke (at 4h and 24h following ischemia) in both groups which underwent ischemia in the lesioned hemisphere compared to Sham group (see Fig 7C, for relative F values and p values), as also observed for *Pmch*. Notably, the SD_IS group displayed an increase of *OxA* over a longer period of time, for 3 days relative to the Sham and IS groups (F (3, 18) = 6.41, p = .003). At 5 and 7 days *OxA* did not show any statistical differences between either groups or hemispheres (Fig 7C).

#### Time course of Orexin/hypocretin receptor-1 (*Ox1R*) and receptor-2 (*Ox2R*)

Since *OxA* binds the *Ox1R* and the *Ox2R* both were assessed to fully understand any involvement of OX-system in the neuroprotection of SD. Interestingly, *Ox1R* was significantly increased during the acute phase of ischemic stroke in the lesioned hemisphere (at 4h to 24 following ischemia) in both groups subjected to ischemia relative to the sham group (see Fig 7C, for relative F values and p values) as already described [34, 35]. Interestingly, this increase was still observed in the SD.IS group only in the contralateral hemisphere at 5 days following interventions compared to the Sham and IS groups (see Fig 7C, for relative F values and p values).

Further, *Ox2R* showed a significant increase in both hemispheres in SD_IS at a unique and early time point (4h) relative to the sham group (F (3, 18) = 10.96, p = .001), while in the IS group this was increased only in the lesioned hemisphere. After this time point *Ox2R* levels remained unaltered between either groups or hemispheres over the time (Fig 7C).

## Discussion

The main results of this study are consistent with data already published showing that SD pre-ischemia is neuroprotective. We also observed that SD pre-ischemia animals had a significant increase of REM sleep during the acute phase of stroke. On the other hand, ischemic animals showed a significant reduction of REM sleep. Finally, an association between ischemic stroke and the beneficial effect of SD with an increase in the gene expression of the MCH and OX systems was found.

### Effects of sleep deprivation on infarct volume

This study confirms the neuroprotective effect of SD pre-ischemia 7 days after stroke [11, 12, 24], which is consistent with data observed at 5 days. In addition, these results show that SD pre-ischemia has a positive effect on infarct volume at 12h. However, this positive effect was not consistent at 24h and 3 days after interventions. This finding was unexpected, and suggests that SD pre-ischemia induces a delay in infarct volume growth, indicating a beneficial effect in addition to the main neuroprotective described so far. One possible explanation is that SD pre-ischemia may prepare the brain to minimize the damage and may promote the formation of new neurons in the chronic phase. This observation is consistent with the notion that preconditioning treatments, such as a short ischemic preconditioning, induce neurogenesis in adult rats’ brains with a maximum peak of cell proliferations after 7 days [36, 37]. Supporting, this interpretation, there is evidence suggesting that sleep itself promotes the production of new cells and neurons [38] while chronic SD causes a reduction of hippocampal cell proliferation and neurogenesis, and consequently may impair hippocampal plasticity and function [39]. Taken together, these data point to the possibility that neurogenesis process is also implicated in the neuroprotective effect elicited by SD although in the present study we did not perform a neurogenesis study based on the administration of BrdU (5-bromo-2-deoxyuridine)

### Alteration in the sleep-wake cycle architecture after ischemia

Our data also confirm that SD pre-ischemia induces a significant increase in the amount of total sleep during the first 24h after ischemia, an effect that persists for 2 days in the SD_IS group following ischemic stroke.

Furthermore, our observations indicate that non-REM sleep also increases during the acute phase of ischemic stroke in all conditions tested, independently of animal treatment, indicating that the early increase of non-REM is not related to an ischemic event or SD treatment, but rather a response to surgical procedures since the ischemic model that we used requires a small craniotomy and/or anesthesia. This observation is consistent with others studies that noticed an effect of isoflurane on sleep homeostasis [40] and non-REM-like EEG activity dominated by slow waves [41]. On the other hand, at 2 days following interventions, non-REM sleep was found to be increased in the SD pre-ischemia animals, and not in other groups investigated including the SD_Sham group. These results suggest that this increase of non-REM sleep at 2 days is not related to SD intervention but rather to an effect of the SD as the preconditioning effect that should be clarified in the future. Indeed, there are studies at supporting the fact that non-REM sleep is a good predictor of favourable outcomes after hemispheric ischemia [17, 18].

Conversely, REM sleep was differently modulated among all conditions from the early phase of ischemic stroke until 2 days after interventions. Indeed, as observed by Mashour and collaborators, 4h of isoflurane anaesthesia does not affect the REM sleep rebound after selective REM sleep deprivation for 24h [42]. Specifically, REM sleep was significantly increased in both groups subjected to SD interventions (SD.IS and SD_Sham groups,) suggesting, that the increases in the intensity of REM sleep, as well as the number of short REM sleep bouts, are related to the extent of sleep deprivation and represent a homeostatic recovery response [43]. Conversely, ischemia animals that did not undergo SD showed a marked decrease of REM sleep as already described previously in both humans and animals [21, 22]. These results support the idea that the increase in the amount of total sleep and particularly the increase of REM sleep during the acute phase of ischemic stroke are induced by previous SD interventions. We suggest that SD pre-ischemia may facilitate the transition from non-REM to REM sleep, probably because the need for sleep increases after previous SD treatment. According to the metabolic hypothesis of sleep, [44], it is possible that sleep change after stroke results from a metabolic recovery of energy stores.

In addition, this study demonstrates that in the early phase of stroke, the increase of REM sleep is negatively correlated with infarct volume assessed after 7 days from ischemic stroke, which suggests a potential role of REM sleep in the neuroprotective effect of SD pre-ischemia. However, the increase of REM sleep in SD pre-ischemia animals might be related to other factors such as reduced inflammation and low level of cytokines in the brains of SD animals [14, 24]. Indeed, REM sleep is particularly influenced by several inflammatory mediators such as cytokines [45, 46], and it has been shown that inflammation strongly exacerbates brain damage in ischemic pathophysiology [47]. Nevertheless, some other mediators like adenosine, which has been described to play a crucial role in the acute preconditioning [48], maybe also implicated in the neuroprotective effect elicited by SD. Although we did not measure adenosine in our study, a body of evidence describe that adenosine plays a role in sleep control [49], and particular the adenosine A1 receptor has been found to be increased following SD in humans [50] and animals [51]. Thus, adenosine influences a set of pathophysiological processes involved in the ischemic stroke, and for some time adenosine and its receptors have been viewed as potential therapeutic targets for the treatment of stroke [48].

### Association of melanin-concentrating hormone (MCH) and Orexin/Hypocretin with the pathophysiology of ischaemic stroke

In the present study, the expression of MCH and OX systems were significantly increased during the acute phase of ischemia (12h and 24h) in both groups that underwent ischemia regardless of SD intervention, indicating that ischemia itself induces the increase of both systems. This data shows that SD pre-ischemia may influence the gene expression of MCH and OX systems for a long period of time (up to 5 days following ischemic stroke). Taken together, these results provide substantial evidence that enables us to consider that these systems are associated with ischemic stroke as well as with the beneficial effect of SD pre-ischemia. In addition, we found that MCH and OX systems were increased in both hemispheres in the acute phase of stroke. This suggests that at early time points the increase of MCH and OX systems were influenced bilaterally i.e. their expression may be modulated by devastating events occurring in the ischemic hemisphere that also influences the contralateral hemisphere. On the contrary, at the late time points their expression are ischemia-related since they were increased exclusively in the ischemic hemisphere.

Our hypothesis regarding the association of these two systems with ischemic stroke is supported by a recent series of clinical and experimental studies describing the involvement of OX-system in post-ischemic stroke. A clinical study on patients with cerebral infarction showed a persistent decrease of cerebrospinal OXA concentrations [52]. Consistent with this finding our results and published experimental studies [34, 53] show that ischemic stroke induces increased expression of *Ox1R* in the brain, which correlates with decreases of OXA in cerebrospinal fluid as an adaptive response to maintain OXA supply to the brain. Finally, a recent study showed that in some conditions such ischemic stroke, cortical neurons may homeostatically switch to the production of OXA as an adaptation mechanism [54]. This observation is consistent with the notion that re-mapping processes that spontaneously occur after stroke [55, 56] may induce an early change of genes expression, and OX and MCH systems may be involved in this process.

However, the mechanism of both the OX and MCH systems in the pathophysiology of ischemic stroke, remain poorly understood and most likely involve inflammation and metabolism systems instead of sleep-wake cycle regulation. Indeed, consistent with our speculation there is a series of studies which have observed that MCH and OX neurons are affected by inflammation, showing a decline when injected with bacterial lipopolysaccharide, which is extensively used to mimic many inflammatory effects of cytokines [57, 58]. It is possible that the increase of both MCH and OX systems in the SD_IS group up until 3 days and 5 days following ischemia may be related to inflammation, which is supposed to be attenuated, compared to IS group alone [14, 24]. Moreover, a study showed that subsets of CD4 (+) T-helper cells in vivo are able to selectively express Pmch [59], which has been known to play a central role in the pathophysiology of ischemic stroke [60].

Taken together, these data point out the possibility that inflammation or metabolic system are the key elements of action of MCH and OX systems [25, 61–63] in the pathophysiology of ischemic stroke as well as in the beneficial effect of SD intervention.

### Strengths, limitations and approaches for future research

This study provides direct evidence that SD pre-ischemia induces a delay in infarct volume growth and that neurogenesis is probably involved. Moreover, we conclude that REM sleep may play a role in neuroprotection, and consequently may facilitate functional recovery after ischemic stroke [12]. To test this hypothesis, we used animals subjected to SD pre-ischemia, which showed an increase of REM sleep as homeostatic sleep response given by previous SD intervention, not inducted artificially by drugs.

One of the limitations of this study is that we only assessed the changes in the mRNA gene expression of the MCH and OX systems, although, it is well established that many post-transcriptional regulations interfere with the level of active protein.

Altogether, these findings provide the basis for further research to understand the role of the MCH system in ischemic stroke and whether MCH also has a neuroprotective effect. Further, to better understand the role of the REM sleep stage in ischemia; a pharmacological treatment with MCH agonists should be performed to prolong REM during the acute phase of stroke, conversely selective REM sleep deprivation should also be performed during the acute phase of stroke.

## Conclusion

Our data indicates that REM sleep may be involved in the neuroprotective effect of SD pre-ischemia, and that both, MCH and orexin systems are increased during the acute phase of stroke. Future studies should assess the role of REM sleep as a prognostic marker, and test MCH and OXA agonists as new treatment options in the acute phase of stroke.

## Supporting Information

S1 Fig **(A)** Not-to-scale representation of the placement of the screw electrodes over the parietal cortex and the cerebellar cortex (pink circles), Ref. = reference and EEG = electroencephalogram. EMG (electromyogram) in grey was bilaterally placed in the neck muscle using wire electrodes **(B)** An example of the homemade-plug used to record EEG/EMG fixed on the head of the animal. **(C)** An example showing how rats were maintained during the EEG/EMG recording. Rats were housed individually in their home cages and then each rat was connected to a flexible cable and swivel (Plastics One) that allowed free movement within the chambers.
(TIF)

S2 Fig Effects of sleep deprivation (SD) pre-ischemia preconditioning on the infarct volume. Lesion volumes uncorrected for edema were calculated by cresyl violet staining at 12 and 24 hours and 3, 5 and 7 days after ischemic surgery are displayed on the x-axis. Infarct volume uncorrected for edema was assessed by multiplying the infarcted area by the slice thickness and combining the volume of the six slices (see methods). Infarct volume (mean ± SEM) were analysed by unpaired t-test (n = 6 per group). Dots represent the infarct volume of each animal during each time point. Asterisks (*) indicate a statistical difference between groups, **p ≤ .01; *** p ≤ .001. These results are consistent with the data shown in the Fig 2 where infarct size was corrected for edema. However, lesion size without correction for edema is overestimated by almost 30% at 24 h and by 20% at 12h of MCAo.
(TIF)

S3 Fig Time course of gene expression of Brain-derived neurotrophic factor (*Bdnf*) in the ischemic and contralateral hemispheres. Time course of gene expression of *Bdnf* at several time points (i.e. 4,12 and 24 hours and 3,4 and 7) after interventions are displayed on the x-axis. Gene expression (mean ± SEM) was assessed by qRT-PCR in rats belonging to the 4 experimental groups: i. SD_IS (n = 6); ii. IS (n = 6); iii. SD_Sham (n = 4); and iv. Sham (n = 6). *Gapdh* was used as the reference gene. The ΔΔCt method was used to determine the fold change in gene expression. Statistical analysis performed by one-way ANOVA did not show any significant changes in the *Bdnf* mRNA expression between groups, and in both hemispheres, at all time points investigated.
(TIF)

S1 Table Data set of the infarct values volume assessed for each animal belonging to the Ischemia group and SD_Ischemia group at several time points (i.e 12h, 24h, 3-5-7 days after interventions). (DOCX)

S2 Table Data set values used to calculate the infarct volume with and without correction for edema. (XLS)

S3 Table Data set of the EEG sleep recording values obtained during the 24 of baseline and after 24 (dark and light phase) 2 and 3 days interventions, for each animal investigated belonging to the 4 experimental groups. (DOCX)

S4 Table Data set of values obtained from the EEG sleep recording expressed as hourly percentage time spent in wakefulness, total amount of sleep (including both non-REM sleep and REM sleep), and non-REM and REM sleep separately across the 24h of baseline (BL) and for the following three days after surgery, for each animal investigated belonging to the 4 experimental groups. (XLSX)

S5 Table Data set of the values used to make a correlation between the amount of total REM sleep phase during the first 24h after ischemic surgery with the infarct volume assessed at 7 days in 12 rats. (DOCX)

S6 Table Data set relative to the gene expression of melanin concentrating hormone (MCH) and orexin-A (OXA) obteined from the ipsilateral and contralateral hemispheres at several time points (i.e 12h, 24h, 3-5-7 days after interventions), in each animal belonging to the 4 experimental groups (Ischemia; SD_Ischemia; Sham and SD_Sham groups). (DOCX)

## Abbreviations

3Vo: three-vessel occlusion method of ischemic stroke
CCA: Common Carotid Artery
cCCA: Contralateral Common Carotid Artery
EEG/EMG: Electroencephalogram/Electromyogram
IS: Ischemia (IS)
iCCA: Ipsilateral Common Carotid Artery
MCA: Middle Cerebral Artery
MCH: Melanin Concentrating Hormone
Mchr1: MCH Receptor 1
Mchr2: MCH Receptor 2
Non-REM sleep: Non-Rapid Eye Movement
OX: Orexin/Hypocretin
OXA: Orexin/Hypocretin A
OXB: Orexin/hypocretin B
OX1R: OX Recepor 1
OX2R: OX Recepor 2
Pmch: Pro-Melanin Concentrating Hormone
qRT-PCR: Quantitative Real-time
REM: Rapid Eye Movement
(rtPA): Recombinant Tissue-type Plasminogen
SD: Sleep Deprivation
SD_IS: Sleep Deprivation followed by ischemia
Sham: Sham surgery
SD_Sham: Sleep Deprivation followed by sham surgery
