# Supplementary figures and images for: Role of REM Sleep, Melanin Concentrating Hormone and Orexin/Hypocretin Systems in the Sleep Deprivation Pre-Ischemia
Source: PLoS One. 2017 Jan 6;12(1):e0168430. doi: 10.1371/journal.pone.0168430 (PMC5218733; doi:10.1371/journal.pone.0168430)

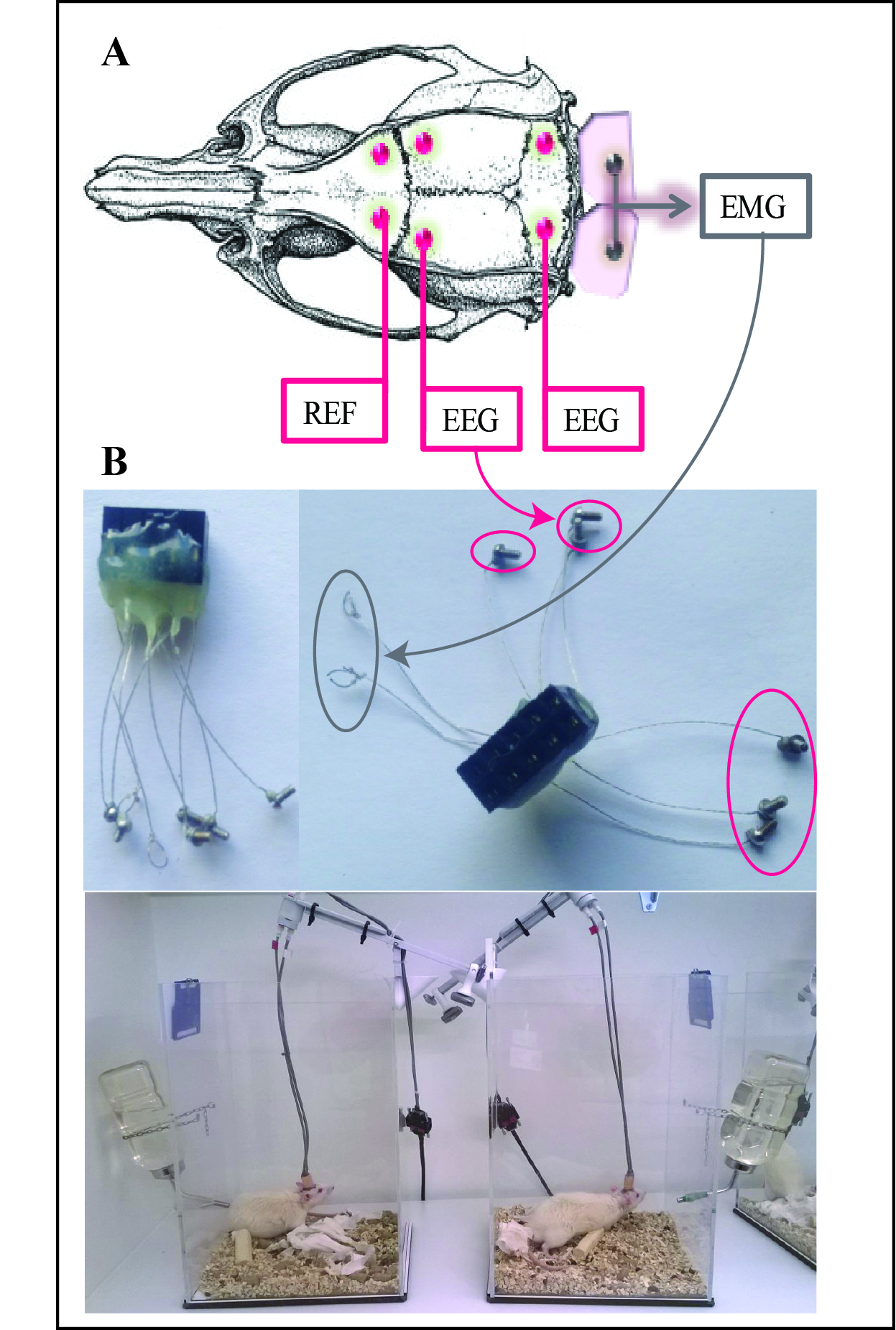

Supplement: S1 Fig — (A) Not-to-scale representation of the placement of the screw electrodes over the parietal cortex and the cerebellar cortex (pink circles), Ref. = reference and EEG = electroencephalogram. EMG (electromyogram) in grey was bilaterally placed in the neck muscle using wire electrodes (B) An example of the homemade-plug used to record EEG/EMG fixed on the head of the animal. (C) An example showing how rats were maintained during the EEG/EMG recording. Rats were housed individually in their home cages and then each rat was connected to a flexible cable and swivel (Plastics One) that allowed free movement within the chambers. (TIF) [file pone.0168430.s001.tif]

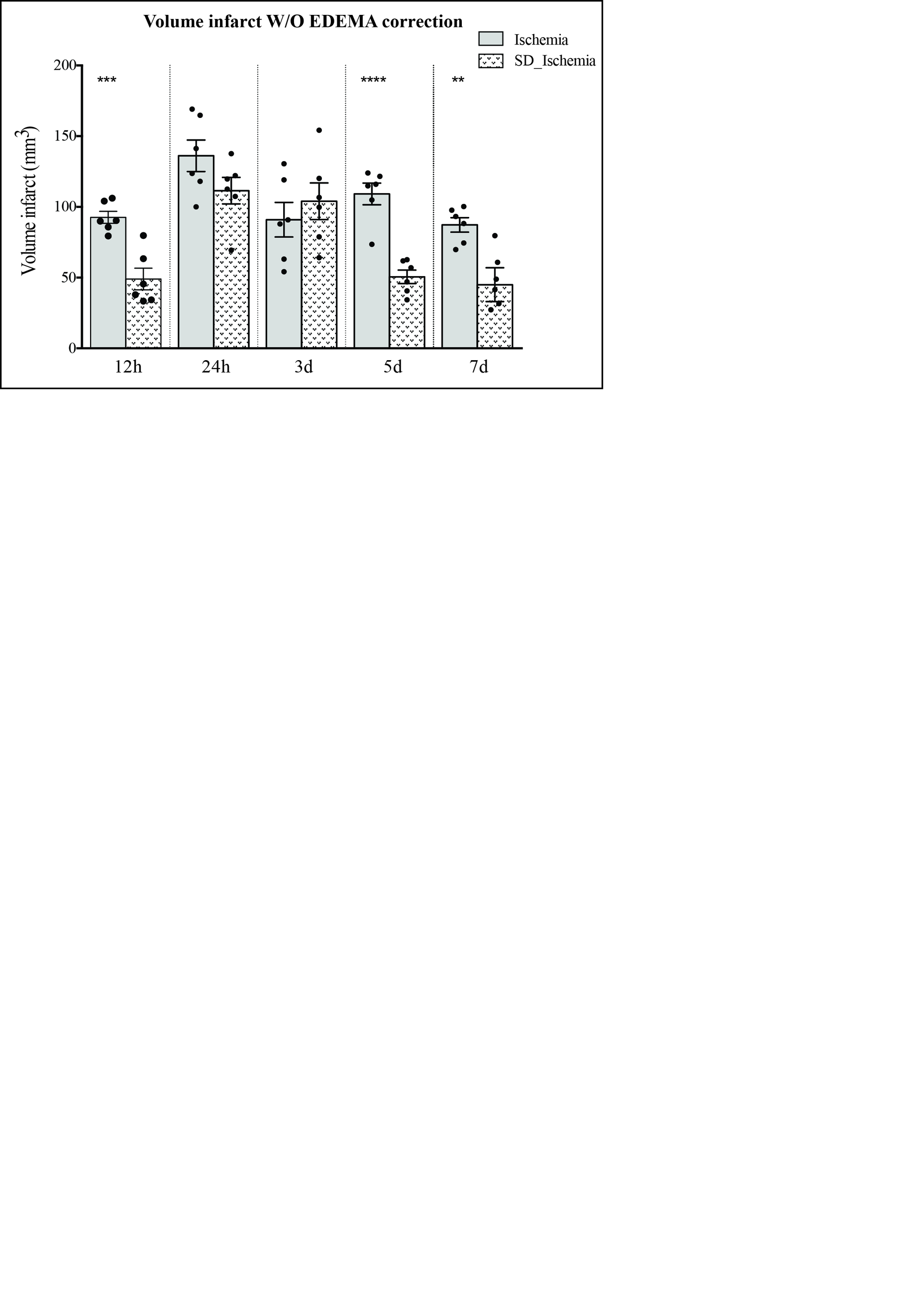

Supplement: S2 Fig — Lesion volumes uncorrected for edema were calculated by cresyl violet staining at 12 and 24 hours and 3, 5 and 7 days after ischemic surgery are displayed on the x-axis. Infarct volume uncorrected for edema was assessed by multiplying the infarcted area by the slice thickness and combining the volume of the six slices (see methods). Infarct volume (mean ± SEM) were analysed by unpaired t-test (n = 6 per group). Dots represent the infarct volume of each animal during each time point. Asterisks (*) indicate a statistical difference between groups, **p ≤ .01; *** p ≤ .001. These results are consistent with the data shown in the Fig 2 where infarct size was corrected for edema. However, lesion size without correction for edema is overestimated by almost 30% at 24 h and by 20% at 12h of MCAo. (TIF) [file pone.0168430.s002.tif]

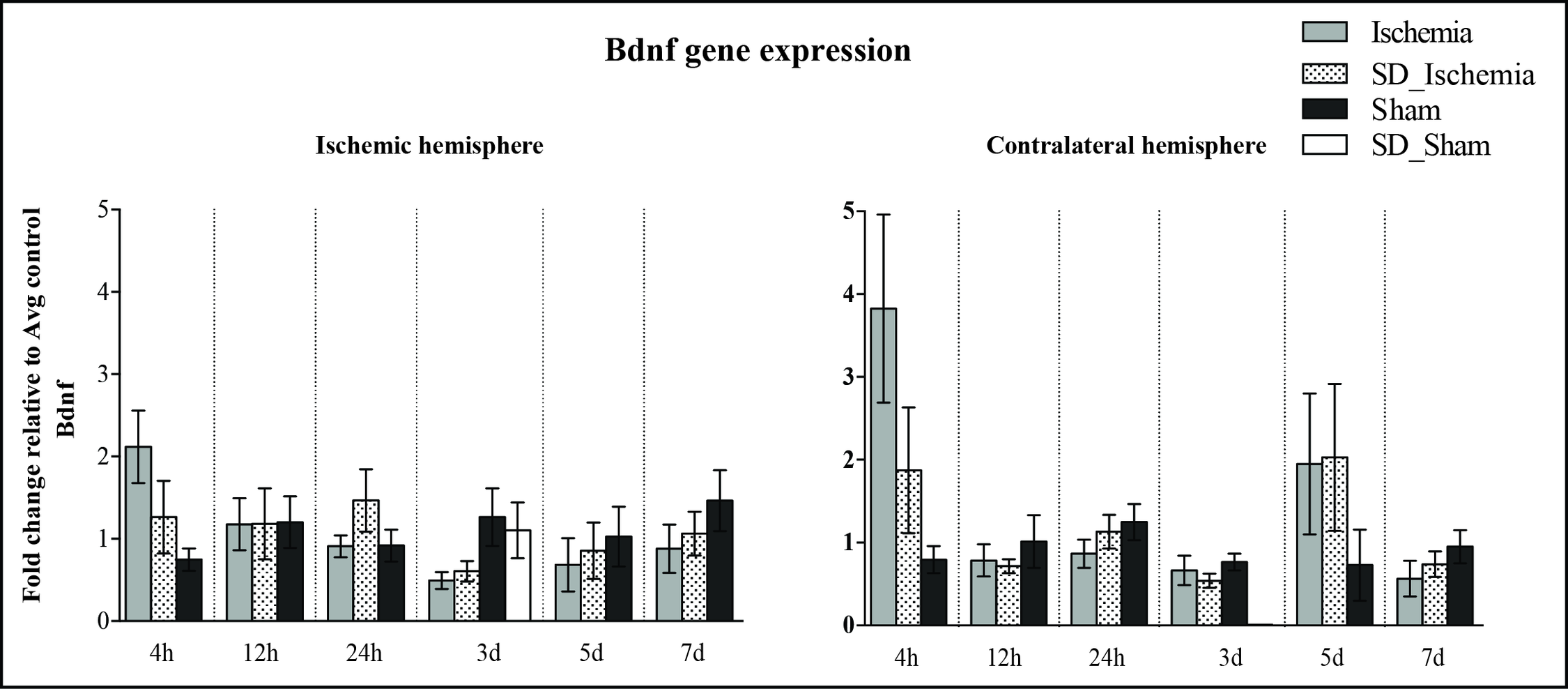

Supplement: S3 Fig — Time course of gene expression of Bdnf at several time points (i.e. 4,12 and 24 hours and 3,4 and 7) after interventions are displayed on the x-axis. Gene expression (mean ± SEM) was assessed by qRT-PCR in rats belonging to the 4 experimental groups: i. SD_IS (n = 6); ii. IS (n = 6); iii. SD_Sham (n = 4); and iv. Sham (n = 6). Gapdh was used as the reference gene. The ΔΔCt method was used to determine the fold change in gene expression. Statistical analysis performed by one-way ANOVA did not show any significant changes in the Bdnf mRNA expression between groups, and in both hemispheres, at all time points investigated. (TIF) [file pone.0168430.s003.tif]
