# Supplementary material for: Role of REM Sleep, Melanin Concentrating Hormone and Orexin/Hypocretin Systems in the Sleep Deprivation Pre-Ischemia
Source: PLoS One. 2017 Jan 6;12(1):e0168430. doi: 10.1371/journal.pone.0168430 (PMC5218733; doi:10.1371/journal.pone.0168430)
Supplement: S3 Table — (DOCX) [file pone.0168430.s006.docx]

**S3 Table** Values for each animal investigated related to EEG sleep recording during the 24 of baseline and after 24 (dark and light phase) 2 and 3 days interventions
