# Supplementary material for: Role of REM Sleep, Melanin Concentrating Hormone and Orexin/Hypocretin Systems in the Sleep Deprivation Pre-Ischemia
Source: PLoS One. 2017 Jan 6;12(1):e0168430. doi: 10.1371/journal.pone.0168430 (PMC5218733; doi:10.1371/journal.pone.0168430)
Supplement: S5 Table — (DOCX) [file pone.0168430.s008.docx]

**S5 Table** Correlation of the amount of total REM sleep phase during the first 24h after ischemic surgery with the infarct volume assessed at 7 days in 12 rats.
