# Supplementary material for: Role of REM Sleep, Melanin Concentrating Hormone and Orexin/Hypocretin Systems in the Sleep Deprivation Pre-Ischemia
Source: PLoS One. 2017 Jan 6;12(1):e0168430. doi: 10.1371/journal.pone.0168430 (PMC5218733; doi:10.1371/journal.pone.0168430)
Supplement: S6 Table — (DOCX) [file pone.0168430.s009.docx]

**S6 Table** Values assessed for each animal belonging to the 4 experimental groups (Ischemia; SD_Ischemia; Sham and SD_Sham groups) for the gene expression of melanin concentrating hormone (MCH) and orexin-A (OXA) in the ipsilateral and contralateral hemispheres at several time points (i.e 12h, 24h, 3-5-7 days after interventions).
